# 

**Published:** 1885-10

**Authors:** 


					﻿• r-	S	*	*
_ »«*-'***
JE JL& S
;■	■.€?'*>■* * '■'	-'4 >-rs-*»3wvra@C
■	®*	• VKeCTfc’
&r-fcS«	a	">v
/	Jsk	m\ ? ■'
.,« m <Tjj( ’'WWHh I ! A s i •.
ffigw RIBW
>’5*^	» h*
Sabs- riptian : *-.00 t> Y<ir in A lraii- r ;	-	-	Si,“fh Vopi-,s.-J5
F. E. DANIEL, M. D., Editor and Publisher, AUSTIN, TEXAS.
___
■ &1&,raS®*’’*-■ St jsel^'Jack *1 <SwS
				

